# Transcriptome profiling of bovine preantral follicles during early folliculogenesis

**DOI:** 10.1186/s40104-026-01407-w

**Published:** 2026-05-14

**Authors:** Noemi Monferini, Pritha Dey, Ludovica Donadini, Filippo Zambelli, Federica Franciosi, Valentina Lodde, Alberto Maria Luciano

**Affiliations:** 1 Reproductive and Developmental Biology Laboratory (ReDBioLab), Department of Veterinary Medicine and Animal Sciences, University of Milan, Via dell’Università 6, Lodi, 26900 Italy; 2 Basic Research Laboratory – Eugin Group, Barcelona, 08006 Spain; 3 Center for Reproductive Biotechnology and Cryobanking, Department of Veterinary Medicine and Animal Sciences, University of Milan, Via dell’Università 6, Lodi, 26900 Italy

**Keywords:** Fertility preservation, Folliculogenesis, Gene expression, Mitochondria, Ovary, Preantral follicles, RAD51, RNA sequencing, Transcriptomics

## Abstract

**Background:**

The ovarian reserve, primarily composed of primordial (PMF), primary (PF), and secondary follicles (SF), holds great potential for fertility preservation, yet remains inaccessible mainly due to the challenges of replicating early folliculogenesis in vitro. The molecular mechanisms governing the activation and differentiation of oocytes from the dormant PMF pool remain poorly defined. In this study, we used a bovine model to investigate early follicular development.

**Results:**

The transcriptome of isolated PMF, PF, and SF revealed different profiles of the follicles during early folliculogenesis. Differential gene expression analyses between PF versus PMF and SF versus PF revealed 689 and 3,206 significantly regulated genes, respectively (FDR < 0.05), highlighting key pathways including PI3K-Akt, Wnt, mTOR, and ECM-receptor interaction. Between PF versus PMF and SF versus PF, 13 and 69 DEGs were identified as transcription factors, respectively. A Likelihood Ratio Test followed by hierarchical clustering identified four major gene expression clusters across the three stages, showing significant enrichment in processes such as ECM-related functions and cell cycle. Based on the enrichment of the cell cycle pathway, we focused on RAD51, which was highly expressed in preantral follicles. Immunohistochemical analysis further showed that RAD51 protein localized in the ooplasm, along with the mitochondrial marker HSP60.

**Conclusion:**

This is the first study to perform bulk RNA sequencing on isolated preantral follicles (PMF, PF, and SF) in cattle. Our findings provide new insights into the stage-specific mechanisms regulating follicle activation and growth in cattle, laying the groundwork for future in vitro fertility preservation strategies in both clinical and conservation contexts.

**Supplementary Information:**

The online version contains supplementary material available at 10.1186/s40104-026-01407-w.

## Introduction

The ovarian reserve, located within the ovarian cortex, consists primarily of primordial follicles (PMF), the earliest stage of preantral follicle development, which constitute the foundation of female reproductive potential [1–3]. In mammals, the establishment of the ovarian reserve occurs during fetal or neonatal development, marking the finite supply of oocytes available throughout life [4, 5]. In humans, the estimated number of follicles at birth ranges from 350,000 to 1,100,000 [6, 7], whereas in cattle, the reserve is markedly smaller, approximately 14,000 to 250,000 follicles [1, 8]. By puberty, extensive follicular atresia and activation reduce the pool to about 100,000 in humans and 84,000 in heifers [1, 2, 8–13]. A marked decline in follicle number in cattle is observed by the fourth year of life [1], paralleling the reduction seen in women from 35 years of age onwards [14, 15].

Despite its apparent abundance, only about 0.1% of the oocytes within the ovarian reserve complete folliculogenesis and are ultimately ovulated, while the remainder undergo atresia through folliculogenesis [10]. Assisted reproductive technologies (ARTs) have expanded access to part of this reserve by enabling the development of gametes from the antral follicle reservoir in vitro [16–19]. Nevertheless, the vast population of preantral follicles, representing over 90% of the ovarian reserve [2, 9], remains largely unexploited, significantly limiting the number of oocytes available for fertility preservation [20]. This is due to the lack of efficient culture systems capable of supporting their growth in vitro. In mice, the in vitro growth of preantral follicles have been achieved and live pups after fertilization has been successfully recapitulated [21, 22]. In humans, multi-step culture systems have yielded a limited number of mature oocytes [23], and recently, a blastocyst was obtained from the in vitro growth of SF [24].

Despite these advances, an efficient and reproducible in vitro folliculogenesis system for higher-order mammals remains elusive, largely due to limited knowledge of follicular differentiation mechanisms and the absence of reliable experimental models for clinical application [10, 25].

Current understanding of folliculogenesis derives primarily from engineered mice, which demonstrate the involvement of signaling pathways, such as PI3K-Akt [26, 27], mTOR [26], Wnt [28, 29], Hippo [30], and Rho-GTPase [31]. However, genetic manipulation is impractical in large mammals, and significant biological differences exist between species, particularly between polytocous species such as mice and monotocous species such as humans and cattle [13, 32–34]. Due to these intrinsic biological differences in follicle formation and growth dynamics, mice are not ideal models for studying folliculogenesis in higher-order mammals [5, 13, 16]. Consequently, bovine species have emerged as a valuable translational model for investigating follicular development and extending in vitro applications to women [35, 36].

To date, most transcriptomic data on folliculogenesis have been conducted in mice [37, 38], sheep [39], cats [40], and humans [41]. In cattle, transcriptomic profiling of oocytes and granulosa cells has been reported starting from the secondary follicle (SF) [42, 43]; however, comparable data for the preceding stages of folliculogenesis are still lacking. Establishing such a dataset is a crucial step toward clarifying the molecular mechanisms governing folliculogenesis and developing more efficient in vitro culture systems for fertility preservation. These advances could enhance reproductive efficiency and genetic gain in cattle herds and support conservation programs for endangered breeds [44].

This study, therefore, aimed to construct and characterize the transcriptomes of bovine PMF, primary (PF), and SF to identify key regulators of follicular transition. Homogeneous populations of mechanically isolated PMF, PF, and SF were subjected to RNA sequencing. The resulting gene expression profiles of each follicle category were then analyzed to establish a reference framework of molecular pathways involved in early follicle differentiation, providing a foundational resource for future studies in ovarian biology and reproductive biotechnology.

## Materials and methods

Unless otherwise stated, all chemicals and reagents used in this study were obtained from Merck Sigma Aldrich, Milan, Italy. Disposable sterile plasticware was purchased from SARSTEDT Srl, Trezzano sul Naviglio (MI), Italy (SARSTEDT Green line for suspension cells) and Thermo Fisher Scientific Inc., Monza (MB), Italy (NUNC IVF Line and Sterilin™). Unless specified otherwise, all procedures were conducted at room temperature (25 °C).

### Ovary collection

Holstein Friesian bovine ovaries were recovered at a local abattoir (IT 2270M CE; Inalca S.p.A., Ospedaletto Lodigiano, LO, Italy) from 12–24 months-old dairy cows subjected to routine veterinary inspection and according to the specific health requirements. No animals were raised or culled to conduct these experiments. Only animals with both ovaries with more than 10 mid-antral follicles (2–8 mm) visible on the ovarian surface [2] were considered. For each animal, ovary pairs were stripped of surrounding fat tissue and ligaments and transferred into a 50-mL tube in sterile saline (NaCl, 9 g/L), supplemented with 100 U/mL penicillin and 0.1 mg/mL streptomycin (Pen/Strep). Ovaries were transported to the laboratory at 4 °C within 1 h and kept cold until processing to minimize ovarian tissue damage [45, 46]. One ovary per animal was randomly selected to conduct the analyses.

### Follicle isolation

Homogeneous populations of PMF, PF, and SF were mechanically isolated, as previously described [3, 9], from 33 animals, in 21 collection experiments. Briefly, one ovarian cortical strip of 20 mm × 10 mm × 0.8 mm, at a time, was minced with a single-edge razor blade, washed in Leibovitz’s L-15 Medium supplemented with 3 mg/mL Bovine Serum Albumin (BSA) and Pen/Strep (isolation medium), and homogenized using IKA ULTRA-TURRAX^®^ T25 Homogenizer (IKA-Werke, Staufen, Germany) with the Dispersing Tool S25D-14G-KS (IKA-Werke, Staufen, Germany) at 3,000 r/min speed for 6 min. The homogenized tissue was passed through a 300-µm mesh strainer (pluriSelect Life Science, Leipzig, Germany) positioned over a 50-mL tube, and each strainer mesh was washed with 5 mL of isolation medium. The filtrate was then serially passed through 100, 70, 40, and 30 µm mesh-size strainers placed atop a 50-mL tube, with each strainer washed with 5 mL of isolation medium. The 70, 40, and 30 µm strainers trapped SF, PF, and PMF, respectively [3]. Follicles were flushed out with 5 mL of isolation medium into a 60-mm Petri dish and collected, based on morphological follicle features [3], under a high-zoom ratio stereomicroscope (Nikon SMZ1270i, Nikon, Tokyo, Japan) using a mouth pipette with a pulled glass capillary (∼100 µm inner diameter). In particular, follicles were classified and subdivided into pools of PMF, wherein the oocyte was surrounded by a single layer of flattened pre-granulosa cells; PF, with one complete layer of cuboidal granulosa cells surrounding the oocyte; and early SF, if the oocyte was surrounded by two or three layers of granulosa cells. To distinguish between early and late SF, we retained only the SF in the 70 µm strainer while excluding those captured on the 100 µm strainer, thus obtaining a more homogeneous follicle population with diameters ranging from approximately 65 to 100 µm. Moreover, considering the reported incidence of atresia affecting approximately 2%–8% of preantral follicles [1, 3, 9, 11, 47], we reasonably assume that the collected population predominantly comprised healthy follicles.

Follicle isolation was completed within 30–45 min and then follicles were placed in a 4-well plate containing 500 µL of αMEM with nucleosides and GlutaMAX™, supplemented with 1 mg/mL bovine serum albumin (fatty acid-free), 1 mg/mL recombinant human insulin, 0.55 mg/mL human transferrin, 0.5 µg/mL sodium selenite, 10−4 IU/mL recombinant human FSH, and Pen/Strep for 1 h of recovery at 38.5 °C with 5% CO_2_ in the air and maximum humidity.

After recovery, each replicate of isolated PMF, PF, and SF was collected in minimum volumes of 0.1% PBS/PVA and stored in 2 mL RNAse-free tubes containing RNA lysis buffer at −80 °C until RNA extraction.

### RNA extraction and sequencing

Isolated PMF, PF, and SF were pooled into distinct follicular classes to create biological replicates for each category. The numbers of PMF, PF, and SF used for RNA extraction and sequencing in each replicate are detailed in Table 1.

**Table 1:** Number of isolated PMF, PF, and SF in each sequenced replicate

Samples	PMF	PF	SF
Replicate 1	119	44	49
Replicate 2	202	49	57
Replicate 3	210	52	54
Replicate 4	-	42	-

### Data processing and differential gene expression analyses

Total RNA from each replicate was extracted using the miRNeasy Tissue/Cells Advanced Micro Kit (Qiagen Srl, Milan, Italy) and eluted in RNase-free water following the manufacturer’s instructions. RNA samples were processed at the Centre of Genomic Regulation, Barcelona, Spain, for library preparation and sequencing. cDNA was generated using SMART-Seq v4 Ultra Low Input RNA kit (Clontech Laboratories, Mountain View, CA, USA). The libraries were prepared using NEBNext^®^ Ultra DNA Library Prep for the Illumina^®^ kit (New England Biolabs, Ipswich, MA, USA) according to the manufacturer's protocol, starting with the cDNA obtained. Library amplification was performed by PCR using NEBNext Multiplex Oligos for Illumina (New England Biolabs, Ipswich, MA, USA). Final libraries were analyzed using an Agilent Bioanalyzer to estimate the quantity. These were then quantified by qPCR using the KAPA Library Quantification Kit (KapaBiosystems, Wilmington, MA, USA). Libraries were sequenced on the Illumina NextSeq2000, generating 50 bp paired-end reads, with a depth of ~35 million paired-end reads per sample.

Raw data were obtained in Fastq format, and read quality was assessed with FastQC (v0.12.0) [48]. Reads were trimmed to remove artificial constructs like adapters, primers, and overcalled polyG sequences using Trimgalore (v0.6.7) [49] and fastp (v0.23.2) [50]. Trimmed reads were mapped to the bovine transcriptome assembly, ARS UCD1.3 (bosTau9), using Salmon (v1.9.0) [51]. Mapped reads were quantified at the transcript level in TPM (transcripts per million) by Salmon. Transcript-level estimates were then summarized to the gene level with the tximport package in Bioconductor/R [52].

All the following analyses were conducted using R programming language and relevant packages. Principal component analysis (PCA) was performed with pcaExplorer (v2.28.0) [53, 54], using all genes. Pairwise differential gene expression (DGE) analyses by Wald’s Test followed by the Benjamini–Hochberg correction were then performed with the DESeq2 (v3.16) [55]. Specifically, for the pairwise comparisons of PF versus PMF and SF versus PF; PMF and PF were set as the reference levels, respectively. Differentially expressed genes (DEGs) with a false discovery rate (FDR) < 0.05 were considered statistically significant and used for downstream analyses. Volcano plots of the DGE analyses were generated with the EnhancedVolcano (v1.24.0) [56]. Raw counts were transformed through the regularized logarithm transformation method with the rlog function of the DESeq2 package, and violin plots for specific genes were generated with ggplot2 (v3.5.1) [57].

### Over representation analyses of differentially expressed genes

Functional enrichment of the DEGs identified in each pairwise comparison was performed via Over Representation Analysis (ORA) for Gene Ontology (GO) and pathways with WebGestalt (v2024) [58]. For GO, Biological Process noRedundant, and for pathways, KEGG, were selected as the functional databases. ORA of all DEGs were performed against the *Bos taurus* species, and the genome protein-coding was set as the reference gene list. The minimum and maximum numbers of genes in the category were set to 5 and 2,000, respectively. The Benjamini–Hochberg correction was used for multiple test adjustments. Biological processes (BP) and KEGG pathways with FDR < 0.05 were considered significant. This analysis was performed on DEGs identified between PF versus PMF and SF versus PF. The results were represented with a lollipop plot created with ggplot2.

### Gene–transcription factor mapping

Transcription factors (TF) influence gene expression by binding to regulatory elements, such as enhancers or silencers, upstream of genes. To further unravel the mechanisms controlling the PMF to PF and PF to SF transitions, the TF of the DEGs, in each pairwise comparison, were mapped by uploading the list of DEGs respective to each pairwise comparison to miRNet 2.0 [59]. miRNet automatically searches for TF targets of genes in specific databases. Here, the TF of the DEGs were identified by searching against *Homo sapiens* via the TRRUST database option on miRNet. A Venn diagram was constructed to visualize the number of DEGs in the input data that were characterized as TF. Genes were then classified into either only DEGs, only TF, or DEGs that were also TF, i.e., common DEGs in the Venn diagram. This analysis was performed on DEGs identified between PF versus PMF and SF versus PF.

### Network analyses and hub gene identification

The networks of DEGs in both comparisons, PF versus PMF and SF versus PF, were constructed through the GeneMANIA plugin [60, 61] on Cytoscape (v3.10.1) [62]. The association data employed was genetic or physical interaction. The network was generated against *Homo sapiens*, with 0 additional related genes and at most 10 attributes, using the default automatically selected weighting method that assigns network weights to maximize the connectivity between all genes using linear regression [63, 64].

The network node (gene) scores were calculated using the CytoNCA plugin [65], which offers different node ranking methods to evaluate the importance of genes (nodes) in a biological network. Hub genes were identified based on local and global-based centrality parameters, and specifically Degree, Betweenness, Eigenvector, and Closeness centrality. Subnetworks of the top 30 hub genes for each centrality measure were generated and merged by intersection to identify the common hub genes and their interactions for the PMF to PF and PF to SF transitions. The individual merged networks identified these genes as potential central hub genes in the respective transitions, PMF to PF and PF to SF.

The interactions of TF mapped to these central hub genes were obtained from miRNet 2.0 in graphml format and imported into Cytoscape. These specific TF were merged by the union to the respective central hub gene networks for PF versus PMF and SF versus PF.

Since the central hub genes are DEGs in the respective pairwise comparisons, these network nodes are shown as rectangles, color-coded according to their log_2_FoldChange (log_2_FC) values, i.e., upregulated DEGs (log_2_FC ≥ 0.05) are color-coded green and downregulated DEGs (log_2_FC ≤ −0.05) are color-coded red. Mapped TF are represented as blue diamonds. To visualize the whole central hub gene-TF network, a Group Attributes Layout was adopted based on the attribute of the gene to be either a central hub gene or a TF.

### Inter-follicle gene expression dynamics and over representation analyses of the clusters

The gene expression dynamics were analyzed using the Likelihood Ratio Test (LRT) of DESeq2 to simultaneously evaluate expression changes across all three follicle stages: PMF, PF, and SF. All genes with FDR < 0.05 were considered significant. The significant genes were then subjected to hierarchical clustering using the degPatterns function of the DEGreport (v1.43.0) [66] to identify clusters of genes with similar expression patterns. Gene abundance was represented as a Z-score. The Z-score is a method for normalizing gene expression, calculated by subtracting the overall average gene abundance from the raw expression for each gene and dividing that result by the standard deviation (SD) of all the measured counts across all samples.

Z-score transformed counts were obtained with DESeq2. The transformed counts of selected genes across the three follicular stages (PMF, PF, and SF) were visualized using a ggplot2 heatmap.

WebGestalt was used to perform an ORA on genes from each generated cluster. KEGG was selected as the functional database for pathway enrichment analysis, performed against the reference gene list of *Bos taurus* protein-coding genes. The minimum and maximum number of genes in a category were set to 5 and 2,000, respectively. The Benjamini–Hochberg correction was used for multiple test adjustments. Pathways with FDR < 0.05 were considered significant. The results were represented with a lollipop plot created with the ggplot2.

### Immunohistochemistry

Ovaries from 6 heifers were transversely cut into 2 to 4 pieces (cross-sections) and fixed in Form-Acetic fixative for 24 h on a rocking plate [3, 67]. Samples were dehydrated in a graded series of ethanol, cleared with xylene, paraffin-embedded, and sectioned at 4 µm for transfer onto SuperFrostPlus slides (ThermoScientific, Milan, IT). Bovine ovarian sections were submitted for the localization of the selected target protein RAD51, with a corresponding negative control performed by omitting the primary antibody. Tissue sections were dewaxed and rehydrated with decreasing ethanol percentages. Antigen retrieval was performed in 10 mmol/L Tris-buffer and 1 mmol/L EDTA (pH 9.00) in thermostatic bath at 98 °C for 25 min. Endogenous peroxidases and unspecific binding sites were blocked sequentially with Peroxidase 1 for 20 min and Background Sniper for 30 min (Biocare Medical, Pacheco, CA, USA). RAD51 primary antibody (Abcam Cat# ab133534, RRID:AB_2722613, dilution 1:200 in PBS) was incubated for 1 h. MACH1 Universal HRP-Polymer Detection (Biocare Medical, Pacheco, CA, USA) was adopted as the secondary antibody. Chromogenic detection was performed with 3,3′-Diaminobenzidine (DAB) (Biocare Medical, Pacheco, CA, USA). After counterstaining with hematoxylin, stained slides were mounted using Bio-Mount HM mounting media (Bio-Optica, Milan, IT). Slides were examined under a light microscope (Nikon Eclipse E600, Nikon) with a Nikon DS-F12 digital camera. Images were captured using the NIS-Elements L image analysis software, with the same camera parameter settings for all acquisitions.

### Immunofluorescence

Tissue samples from the ovaries of heifers (*n* = 6) were processed and mounted on slides as described above. Bovine ovarian sections were processed, including antigen retrieval, as previously described. After antigen retrieval, endogenous peroxidase activity was blocked with 3% H_2_O_2_ for 20 min, and nonspecific binding sites were blocked by incubating sections in PBS containing 10% fetal bovine serum, 0.05% Tween-20, and 3% bovine serum albumin (BSA) for 1 h. The primary antibodies RAD51 (Abcam Cat# ab133534, RRID:AB_2722613, dilution 1:200 in PBS 0.05% Tween with 1% of BSA), with ɣH2AX (Sigma-Aldrich Cat# 05-636, RRID:AB_309864, dilution 1:100 in PBS 0.05% Tween with 1% of BSA) or HSP60 (NeoBiotechnologies Cat# 3329-MSM4-P0, dilution 1:100 in PBS 0.05% Tween with 1% of BSA) were co-incubated for 1 h at room temperature. After washing, sections were covered with a Rhodamine (TRITC)-conjugated Donkey Anti-Rabbit IgG (Jackson ImmunoResearch Labs Cat# 711-025-152, RRID:AB_2340588, dilution 1:100 in PBS 0.05% Tween with 1% of BSA) and an AlexaFluor™ 488-conjugated Donkey Anti-Mouse IgG (Molecular Probes Cat# A-21202, RRID:AB_141607, dilution 1:500 in PBS 0.05% Tween with 1% of BSA), then incubated at room temperature for 30 min. Negative controls were performed by omitting each primary antibody individually while retaining both secondary antibodies. Slides were then mounted with Vectashield® Antifade Mounting Medium with DAPI (Vector Laboratories, Newark, CA, USA) and examined under an epifluorescence microscope (Nikon Eclipse E600, Nikon) with a Nikon DS-F12 digital camera. Images were captured using the NIS-Elements L image analysis software, with the same camera parameter settings for all acquisitions.

### Statistical analysis

All analyses were performed in R (v4.3.0) using standard bioinformatics packages. RNA-seq count data were analyzed with DESeq2 (v3.16); counts were normalized using the TPM method, and differential expression was assessed via the Wald test. The gene expression dynamics were analyzed using the Likelihood Ratio Test (LRT) of DESeq2 and hierarchical clustering was performed using the degPatterns function of the DEGreport (v1.43.0). False discovery rate (FDR) correction was applied using the Benjamini–Hochberg procedure, with significance defined as FDR < 0.05.

## Results

### DGE and ORA analyses identified biological processes and pathways enriched between consecutive follicular stages

**Fig. 1:**
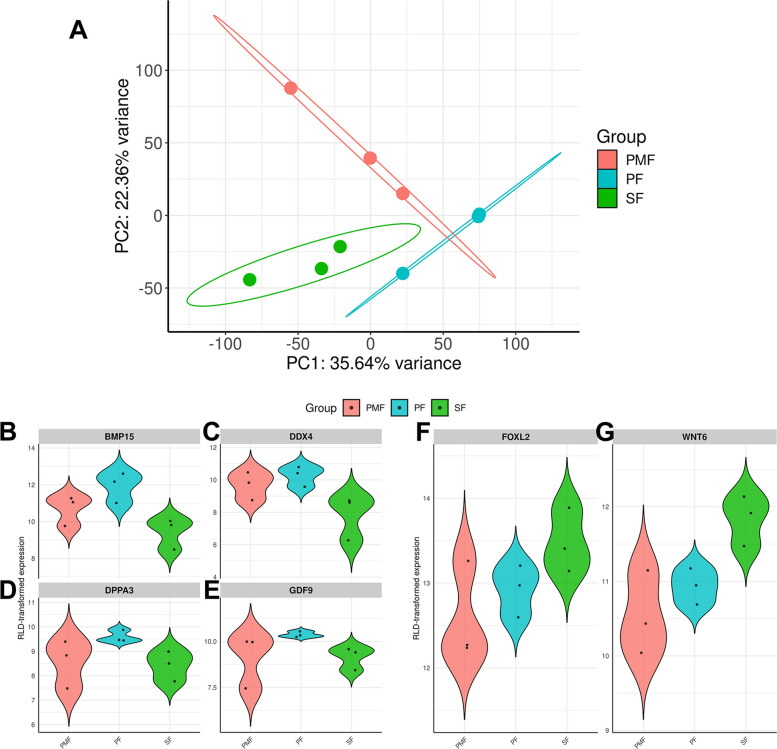
PCA Plot and control genes expression. **A** PCA plot of the transcriptome of RNA-seq data from follicle replicates (*n* = 9) at different stages of development, subdivided into homogeneous populations of PMF (*n*=3), PF (*n*=3), and SF (*n*=3). The PCA was performed on all the genes and showed clear clustering of the samples. Violin plots of control genes specific to oocyte (**B–E**) and granulosa cells (**F**, **G**) in the three follicle classes were reported as regularized log-transformed counts (rlog) obtained from DESeq2

The mapping rate ranged from 78% to 91% across all samples. One of the four PF replicates did not cluster with the other three and was therefore considered an outlier and removed from further analysis. The PCA plot with all the samples is reported in Additional file 1. After outlier removal, the resulting PCA plot showed that the PMF, PF, and SF groups exhibited distinct transcriptome profiles (Fig. 1A). Although follicle compartment (oocyte and granulosa cell) isolation and sequencing were not performed in this study, we noted a similar pattern of expression of oocyte-specific (*BMP15*,* DDX4*,* DPPA3*, and *GDF9*) and granulosa cell-specific (*FOXL2* and *WNT6*) genes across the preantral follicle stages, as evidenced in Fig. 1B–E and F–G, respectively.

**Fig. 2:**
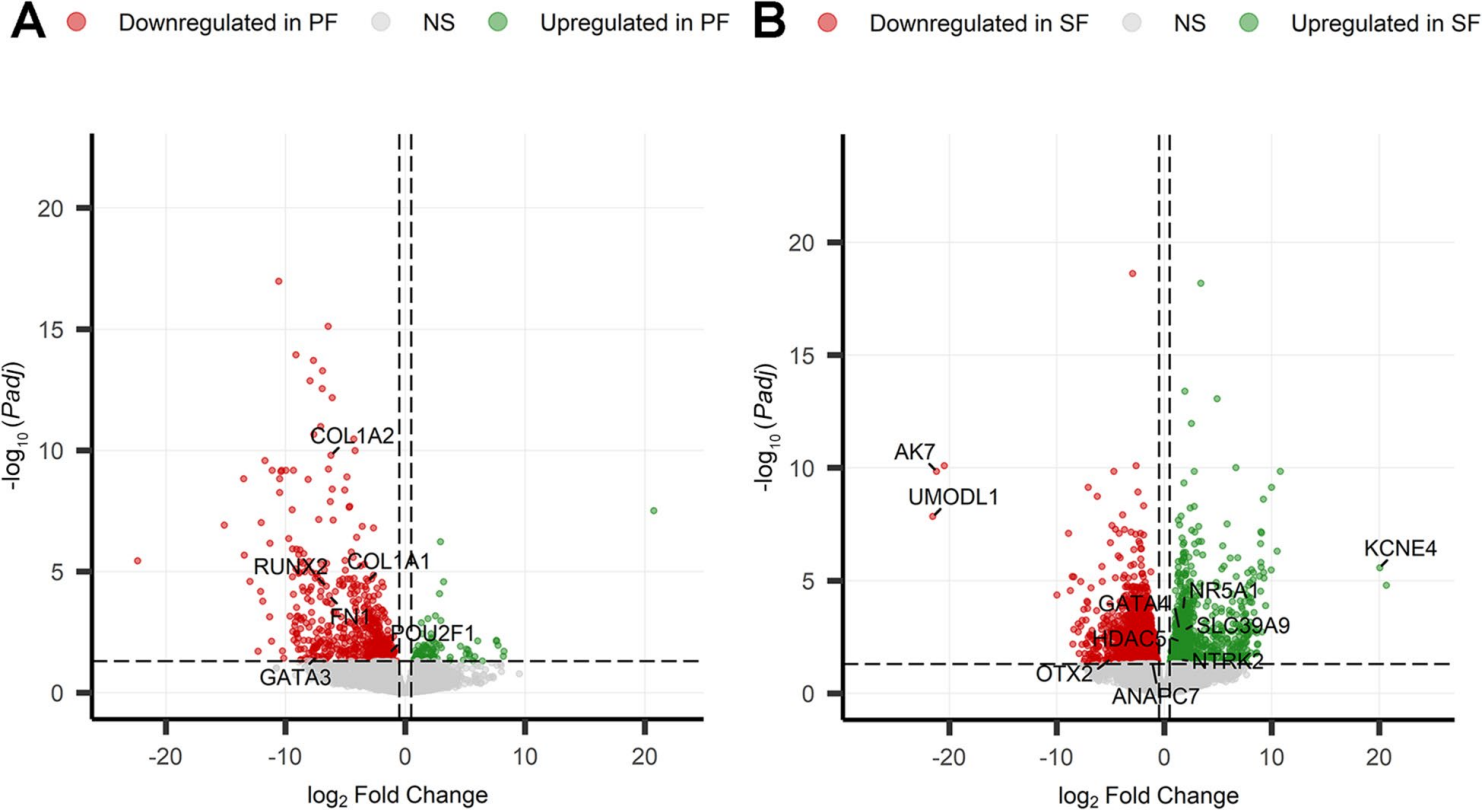
DGE analyses PF versus PMF and SF versus PF. DGE analyses revealed 689 and 3,206 statistically significant DEGs (FDR<0.05) in PF versus PMF (**A**) and SF versus PF (**B**), respectively. In the volcano plot, red dots represent significantly downregulated genes and green dots represent significantly upregulated genes. Gray dots indicate non-significant genes. The *x*-axis represents the log_2_FoldChange, and the *y*-axis represents −log_10_Padj, i.e., the FDR (adjusted *P*-value)

In total, 689 and 3,206 statistically significant DEGs (FDR < 0.05) were screened from the pairwise comparison between PF versus PMF and SF versus PF groups, respectively (Fig. 2A and B).

In the PF versus PMF pairwise comparison, more genes were downregulated in PF (nDEGs = 595) compared to those upregulated (nDEGs = 94) (Fig. 2A), while in the SF versus PF pairwise comparison, more genes were downregulated in SF (nDEGs = 1,935) compared to those upregulated in SF (nDEGs = 1,271) (Fig. 2B). Considering the low transcriptional activity of PMF [68], more follicles were sequenced compared to PF and SF. The complete list of DEGs was reported in Additional file 2—Table S1A and S1D.

**Fig. 3:**
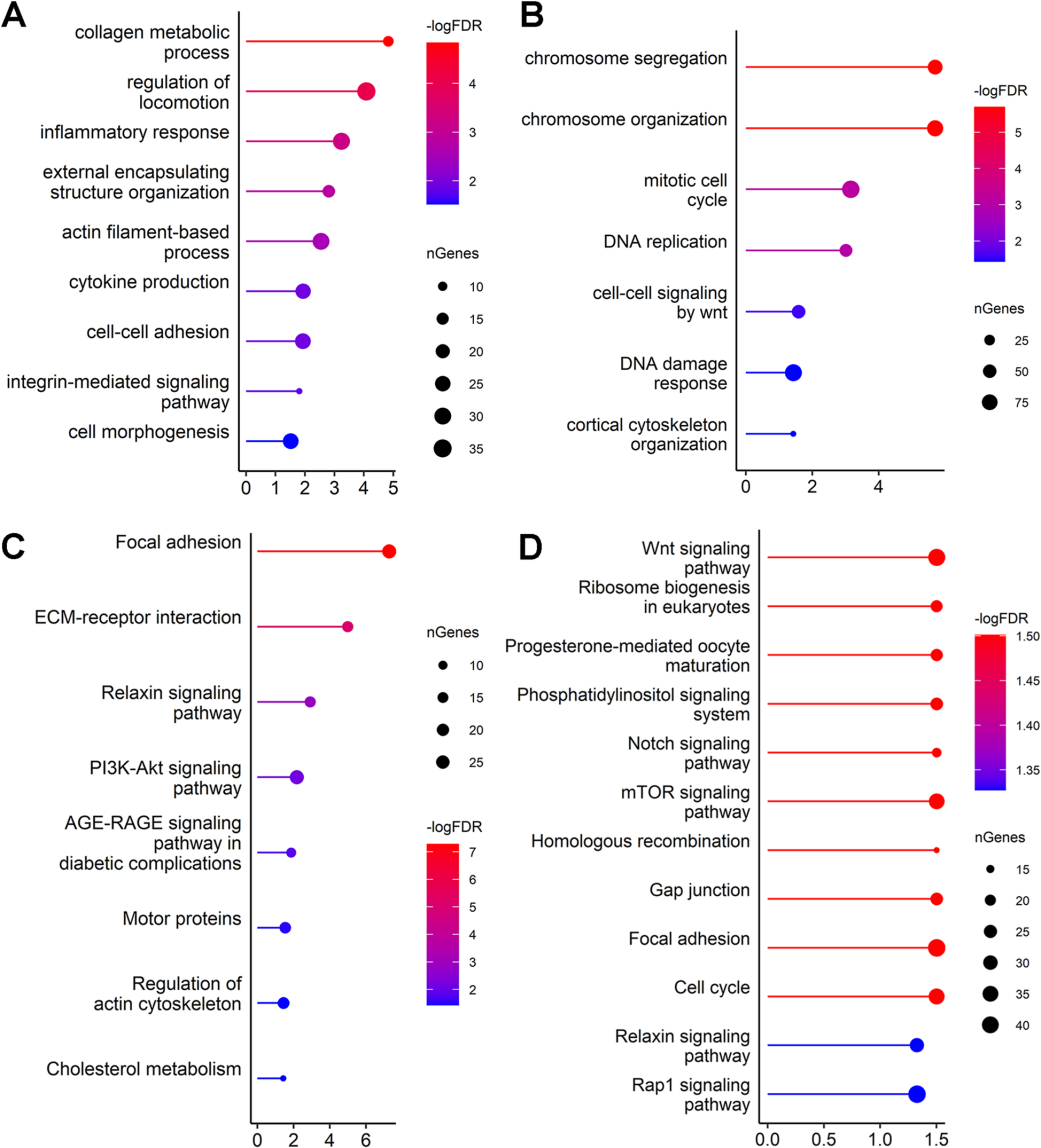
ORA of PF versus PMF and SF versus PF. Selected enriched GO terms BP (**A**) and KEGG pathways (**B**) of DEGs in the PF versus PMF comparison. Selected enriched GO terms BP (**C**) and (**D**) KEGG pathways of DEGs in the SF versus PF comparison. Significantly enriched BP and pathways (FDR < 0.05) are represented with their log-transformed significance (−log_10_ FDR) on the *x*-axis. Higher values (red) indicate greater statistical significance, while less significant processes are shown in blue. The size of each point corresponds to the number of DEGs from the respective pairwise comparisons that overlap with the gene set described for each BP or pathway

ORA was performed on all DEGs in each pairwise comparison to obtain a comprehensive overview of the top 50 functionally enriched BP and KEGG pathways, and only the most relevant based on follicle biology were reported in Fig. 3. The most relevant functionally enriched BP and pathways in PF versus PMF are illustrated in Fig. 3A and B, and the most relevant BP and pathways for SF versus PF in Fig. 3C and D. All significant (FDR < 0.05) BP and KEGG pathways obtained from the ORA of the DEGs between PF versus PMF and SF versus PF are listed in Additional file 2—Table S1B, S1C, S1E, and S1F.

### Gene–transcription factor mapping highlight stage-specific transcriptional regulation

**Fig. 4:**
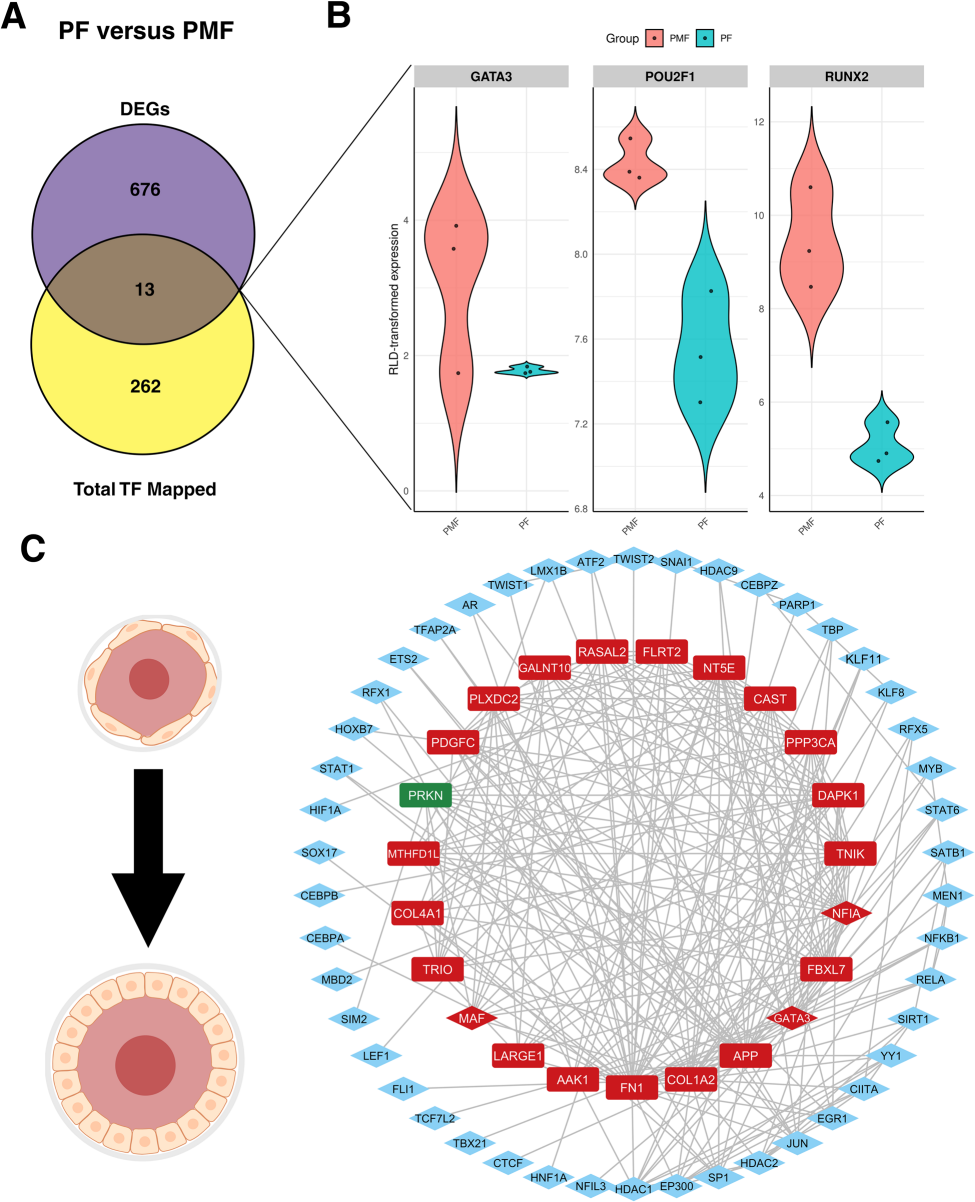
Mapped TF and hub genes in PF versus PMF. **A** Venn diagram of the total number of TF mapped through miRNet by the TRRUST database and the DEGs between PF and PMF. **B** Violin plots of characterized TF *GATA3*, *POU2F1* and *RUNX2*. **C** Central hub genes were identified putatively regulating the PMF to PF transition. Genes shown in rectangles are DEGs but not TF. Genes in blue diamond shapes are TF and not DEGs. Genes in red diamond shapes are both DEGs and TF. DEGs and TF expressed in the PF-versus-DGE analysis wherein PMF is the reference level or ‘control’ are color-coded as follows: green indicates gene upregulation, and red indicates downregulation

TF regulating DEGs in both pairwise comparisons were obtained by mapping the genes to miRNet. miRNet (TRRUST database) identified 274 TF that may regulate the 689 DEGs between PMF and PF. Of those, 12 TF were found to be downregulated in PF compared to PMF in this dataset, and only *PRKN* was upregulated (Fig. 4A). The violin plots in Fig. 4B show the expression of some of the mapped TF *GATA3*, *POU2F1* and *RUNX2*. The latter, has previously been identified to be important in determining granulosa cell fate [69].

**Fig. 5:**
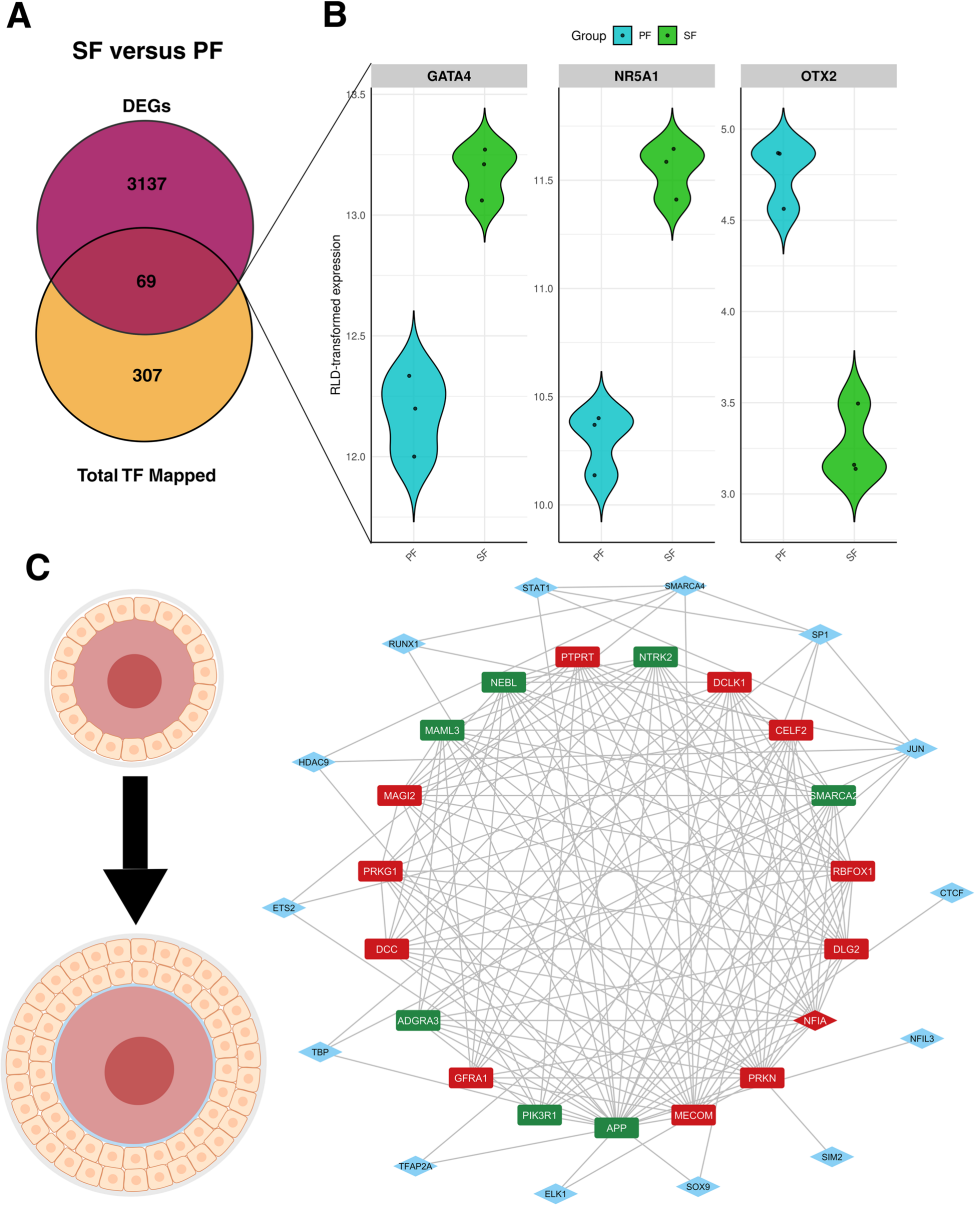
Mapped TF and hub genes in SF versus PF. **A** Venn diagram of the total number of TF mapped through miRNet by TRRUST database and the DEGs between SF and PF. **B** Violin plots of characterized TF *GATA4, NR5A1,* and *OTX2*. **C** Central hub genes identified that may regulate the PF to SF transition. Genes shown in rectangles are DEGs but not TF. Genes in blue diamond shapes are TF and not DEGs. Genes in red diamond shapes are both DEGs and TF. DEGs and TF expressed in the SF versus PF DGE analysis, wherein PF is the reference level or ‘control’, are color-coded as follows. Green indicates gene upregulation, and red indicates downregulation

Similarly, a total of 375 unique TF were identified during the PF to SF transition, of which 69 overlapped with the 3,206 DEGs between PF and SF (Fig. 5A). We also charted the expression of TF *GATA4, NR5A1* and *OTX2* in Fig. 5B. All TF mapped in both pairwise comparisons are listed in Additional file 3—Table S2A and S2B.

### Network analyses and hub gene identification

Constructing a network can help understand the molecular machinery governing the biological states of PMF, PF, or SF. Respective networks of the DEGs between PF versus PMF and SF versus PF were generated (Additional file 4—Fig. S2 and S3). Upon merging the top 30 hub genes across different centrality measures, 23 and 19 potential central hub genes that may play a role in the PMF to PF and PF to SF transitions, respectively, were found. Of the 49 TF (Fig. 4C blue and red diamond-shaped nodes) regulating the central hub genes in the PMF to PF transition, *GATA3* (red diamond-shaped node), *MAF* (MAF BZIP transcription factor) and *NFIA* (nuclear factor 1 A-type) were expressed in this dataset and were identified as potential central hub genes guiding this transition. *GATA3, MAF *and* NFIA* were upregulated in PMF in comparison to PF (Fig. 4C). Other 20 non-TF hub genes include *AAK1* (AP2 associated kinase 1), *APP* (Amyloid-beta precursor protein), *CAST* (calpastatin), *COL1A2* (collagen type I alpha 2 chain), *COL4A1* (collagen type IV alpha 1 chain), *DAPK1* (death associated protein kinase 1), *FBXL7* (F-Box and leucine rich repeat protein 7), *FLRT2* (fibronectin leucine rich transmembrane protein 2), *FN1* (fibronectin), *GALNT10* (polypeptide N-acetylgalactosaminyltransferase 10), *LARGE1* (LARGE xylosyl- and glucuronyltransferase 1), *MTHFD1L* (methylenetetrahydrofolate dehydrogenase (NADP+ dependent) 1 like), *NT5E* (5'-nucleotidase Ecto), *PDGFC* (platelet derived growth factor C), *PLXDC2* (plexin domain containing 2), *PPP3CA* (protein phosphatase 3 catalytic subunit alpha), *PRKN* (Parkin RBR E3 ubiquitin protein ligase), *RASAL2* (RAS protein activator like 2), *TNIK* (TRAF2 and NCK interacting kinase), *TRIO* (Trio Rho guanine nucleotide exchange factor).

Similarly, during PF to SF transitions, 15 TF (Fig. 5C blue diamond-shaped nodes) were identified that may regulate the central hub genes. Only 1 TF (NFIA) was identified as a potential central hub gene; which along with the mapped TF, shown as blue diamond-shaped nodes in Fig. 5C, may collectively regulate the shortlisted candidate central hub genes in the PF to SF transition, including *ADGRA3* (adhesion G protein-coupled receptor A3), *APP* (amyloid beta precursor protein), *CELF2* (CUGBP Elav-like family member 2), *DCC* (DCC netrin 1 receptor), *DCLK1* (doublecortin like kinase 1), *DLG2* (discs large MAGUK scaffold protein 2), *GFRA1* (GDNF family receptor alpha 1), *MAGI2* (membrane associated guanylate kinase, WW and PDZ domain containing 2), *MAML3* (mastermind like transcriptional coactivator 3), *MECOM* (MDS1 and EVI1 complex locus), *NEBL* (nebulette), *NTRK2* (neurotrophic receptor tyrosine kinase), *PIK3R1* (phosphoinositide-3-kinase regulatory subunit 1), *PRKG1* (protein kinase cGMP-dependent 1), *PRKN*, *PTPRT* (protein tyrosine phosphatase receptor type T), *RBFOX1* (RNA binding fox-1 homolog 1), *SMARCA2* (SWI/SNF related BAF chromatin remodeling complex subunit ATPase 2).

These TF and DEGs may be critical in transitioning from PMF to PF and PF to SF in the bovine ovary. Collectively, these genes contribute to elucidating the genetic repertoire that influences the PMF, PF, and SF, and may act as potential candidate biomarkers to monitor during the PMF to PF and PF to SF transitions.

### Gene expression dynamics across preantral follicles point to conserved enriched pathways

**Fig. 6:**
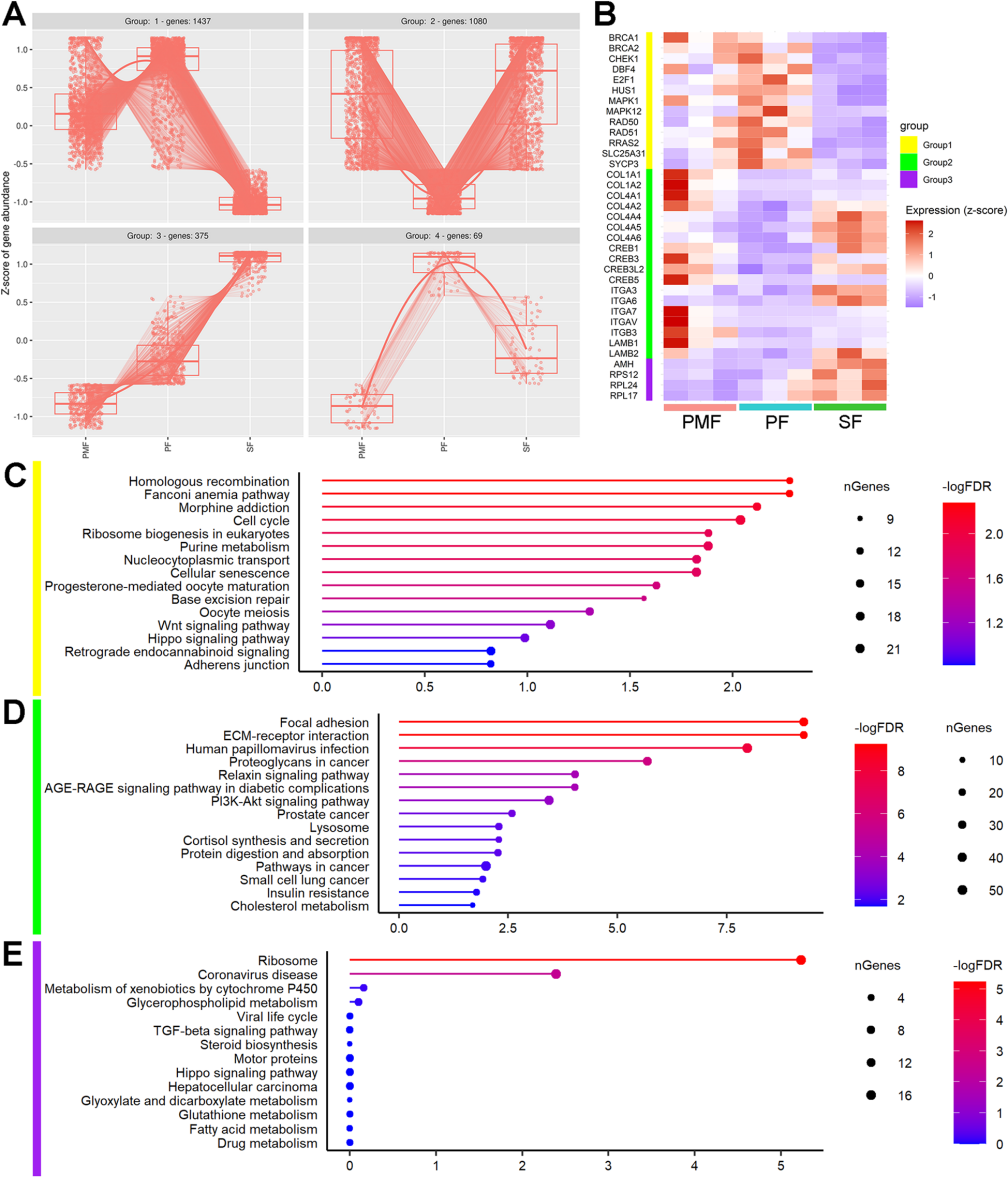
Gene expression dynamics along PMF, PF, and SF. **A** Inter-follicle time course gene expression analysis identified significant genes (FDR<0.05) with similar patterns of expression, which were hierarchically clustered into 4 groups. Each group has a different expression pattern based on the expression of the clustered genes at each stage of follicular development. The number of genes for each group is noted on the header of the corresponding graph. Gene abundance is indicated on the *y*-axis and expressed as a Z-score. **B** The expression of selected genes across the three replicates of PMF, PF, and SF is reported in the heatmap, with values expressed as Z-scores. Red indicates higher expression, while blue indicates lower expression. The vertical bar identifies the group in which the genes were identified as: yellow for Group 1, green for Group 2, and purple for Group 3. **C–E** Top 15 enriched KEGG pathways of Group 1, Group 2, and Group 3 genes, respectively. Enriched pathways (FDR<0.05) are represented with their log-transformed significance (−log_10_FDR) on the *x*-axis. Higher values (red) indicate greater statistical significance, while less significant pathways are shown in blue. The size of each point corresponds to the number of genes in the respective clusters/groups that overlap with the gene set described for each specific KEGG pathway

To simultaneously elucidate gene expression dynamics across the follicular stages, we conducted an inter-follicle gene expression analysis, revealing 2,961 significant genes. Figure 6 shows the gene expression dynamics of PMF, PF, and SF across four groups generated by hierarchical clustering. Groups 1 and 2 had 1,437 and 1,080 genes, respectively, while groups 3 and 4 had 375 and 69 genes, respectively. The four groups show distinct expression patterns across the three follicular stages.

During the transition from PMF to PF, gene expression in Group 1 increases, then decreases during the transition from PF to SF (Fig. 6A). Functional enrichment analysis of Group 1 (Fig. 6C) showed a significant over-representation of pathways such as homologous recombination (FDR = 0.005), cell cycle (FDR = 0.009), ribosome biogenesis in eukaryotes (FDR = 0.013), nucleocytoplasmic transport (FDR = 0.015), cellular senescence (FDR = 0.015), progesterone-mediated oocyte maturation (FDR = 0.024) and oocyte meiosis (FDR = 0.049).

Homologous recombination (HR) is the most significantly enriched pathway in Group 1; its genes are involved in double-strand break (DSB) repair mechanisms, such as *RAD50*,* RAD51*,* BRCA1*,* BRCA2*, and *SYCP3* [70]. Other pathways, such as the cell cycle and cellular senescence, involve key genes that regulate cell cycle progression, including checkpoint genes such as *CHEK1*, which control cell proliferation in response to DNA damage or unreplicated DNA [71, 72]. Additionally, other genes participate in cell cycle control, such as *E2F1*,* HUS1*, and *SLC25A31*, as well as genes that positively regulate the cell cycle, such as *DBF4*,* MAPK1*, and *MAPK12* (which are regulated by *RRAS2*) [73–75]. All these genes were downregulated in SF compared to PF, and they did not show differences in the PF versus PMF comparison, except for *MAPK12*, which was upregulated in PF (Fig. 6B).

Group 2 genes showed a descending trend of expression during the transition from PMF to PF and an ascending trend from PF to SF (Fig. 6A). Significant enrichment revealed cell communication and developmental pathways such as focal adhesion (FDR = 5.560e-10), ECM-receptor interaction (FDR = 5.560e-10), relaxin signaling pathway (FDR = 9.315e-05), AGE-RAGE signaling pathway in diabetic complications (FDR = 9.315e-05), PI3K-Akt signaling pathway (FDR = 3.614e-04), lysosome (FDR = 0.005), gap junction (FDR = 0.045), and regulation of actin cytoskeleton (FDR = 0.022) (Fig. 6D).

The genes *COL1A1*,* COL1A2*,* COL4A1*,* COL4A2*,* COL4A4*,* COL4A5*, and *COL4A6* are commonly associated with focal adhesion, ECM-receptor interaction, the relaxin signaling pathway, the AGE-RAGE signaling pathway in diabetic complications, and the PI3K-Akt signaling pathway. In particular, Collagen I was downregulated from PMF to PF, and Collagen IV was upregulated from PF to SF (Fig. 6B). The expression patterns of Group 2 genes involved in focal adhesion and ECM-receptor interactions highlight laminins, collagens, and integrins. Additionally, downstream genes, such as the *CREB* family, act as transcription factors to promote cell proliferation and PMF activation through the PI3K-Akt and relaxin signaling pathways [76, 77].

The linear increase in the expression of genes in Group 3 showed significant enrichment of processes related to protein synthesis, i.e., ribosome (FDR = 5.863e-06) (Fig. 6E). Although the TGF-beta pathway was not statistically significant, *AMH* (anti-Müllerian hormone) increased during follicle development, with a significant upregulation at the SF stage (Fig. 6B), consistent with the well-known pattern of expression of this gene. The number of genes in Group 4 was too low to identify relevant pathways.

### Protein localization of RAD51 reveals expression in preantral follicle oocytes and potential mitochondrial link

Given the central role of *RAD51* in DNA repair mechanisms and its tight coordination with cell-cycle progression observed in Group 1, we next sought to determine where RAD51 is localized within preantral follicles.

**Fig. 7:**
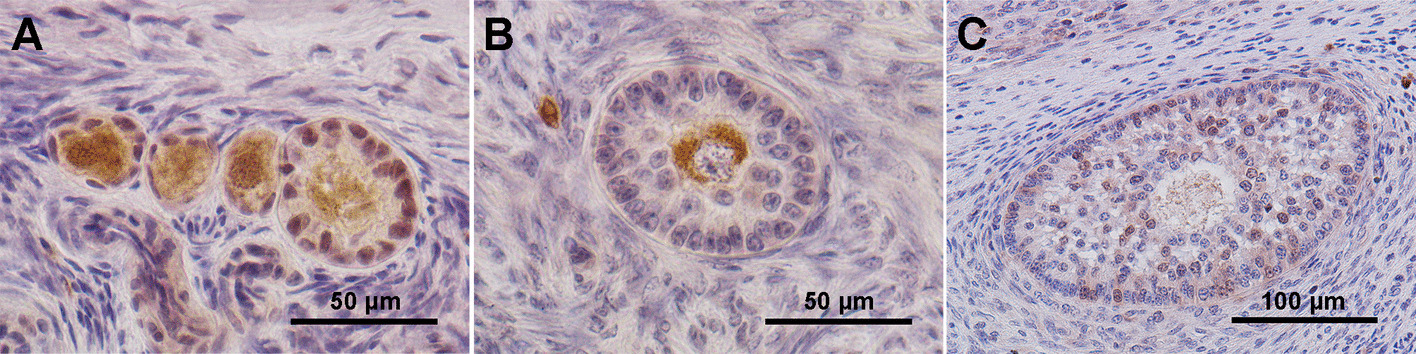
Immunohistochemistry of RAD51 in bright field. Representative pictures of RAD51 localization in PMF and PF (**A**), early SF (**B**), and late SF (**C**). Scale bar 50 µm (**A** and **B**); scale bar 100 µm (**C**)

Immunohistochemistry of ovarian tissue sections was performed and evaluated under brightfield microscopy. Intense staining with RAD51 antibody was detected in the oocytes and GCs of PMF and PF (Fig. 7A). It was lowest in SF, where it was retained by the oocyte but faint in the GCs (Fig. 7B). A weak signal was visible only in the GCs of more advanced stage of folliculogenesis (late SF) (Fig. 7C). Control sections showed no detectable signal, confirming staining specificity (Additional file 5—Fig. S4).

**Fig. 8:**
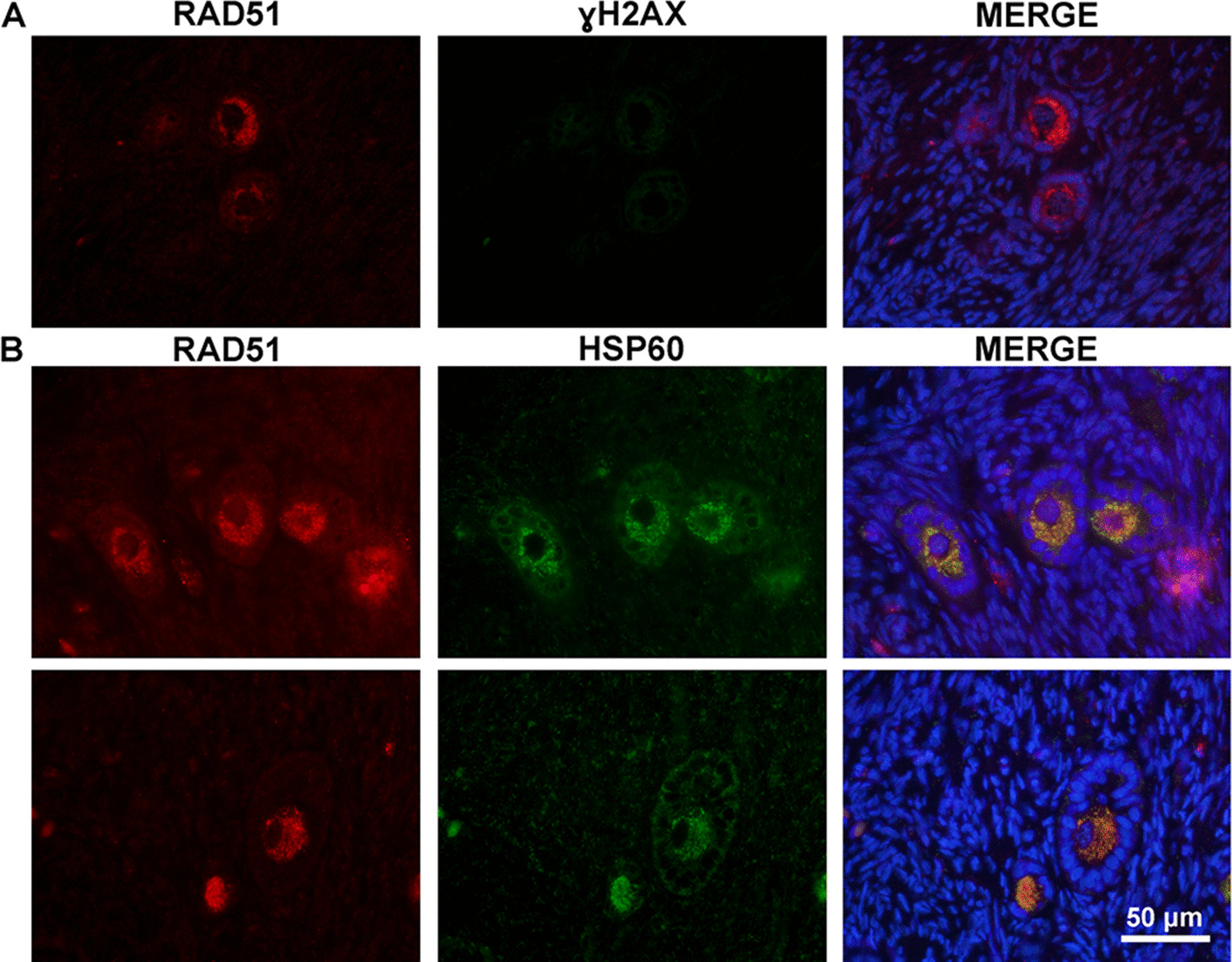
Immunofluorescence of RAD51 colocalized with ɣH2AX or HSP60. Representative picture of the localization of RAD51 (red) with ɣH2AX (green) (**A**) and of RAD51 (red) with HSP60 (green) (**B**). DAPI = blue. Scale bar 50 µm

To investigate whether RAD51 was associated with DNA DSB, immunostaining with fluorescent secondary antibodies was performed to assess its colocalization with ɣH2AX, a well-known marker of DNA damage. No RAD51 signal was detected in the oocyte nuclei of PMF, PF, and SF (Fig. 8A). Instead, RAD51 was predominantly detected in the cytoplasm. To explore its localization, a double immunofluorescence with HSP60, a mitochondrial marker, was conducted. Both RAD51 and HSP60 signals localized in the cytoplasm of PMF, PF, and SF oocytes (Fig. 8B). Negative controls for RAD51-ɣH2AX and RAD51-HSP60 double staining showed no detectable fluorescence, further confirming staining specificity (Additional file 5—Fig. S5).

## Discussion

This study provides the first comprehensive transcriptomic characterization of the early stages of bovine folliculogenesis, revealing unique molecular features underlying the transitions from PMF to SF.

During the PMF to PF transition, enrichment of pathways associated with actin organization, integrin signaling, and cellular adhesion molecules indicates changes in cell contact and morphology, which are essential for follicle activation and transition [10, 78, 79]. This observation is reinforced by the enrichment of the integrin-mediated signaling pathway [80], together with the actin filament-based process, cell–cell adhesion, participating in the cell sensing and response [81, 82].

Interestingly, there were significantly more downregulated genes and pathways in the PF versus PMF comparison, which may be attributable to the larger PMF population sequenced, which in turn may influence relative gene abundance [41]. However, as shown in Fig. 1, the distribution of oocyte- and GC-specific genes after DESeq2 normalization appears fairly similar across stages, suggesting that differences in number of follicles in each replicate and total cell numbers have a limited effect on the observed expression patterns. The higher number of regulated genes at the PMF stage may then reflect functional mechanisms involved in the maintenance of follicle quiescence [83]. In this context, key pathways downregulated during the PF versus PMF comparison, such as focal adhesion, ECM-receptor interaction, and the PI3K-Akt signaling pathway, may help maintain the balance between PMF quiescence and activation [84–86]. Quiescence maintenance can be linked to a physiological deposition of collagen fibers in the PMF ECM, a process provoked by the accumulation of advanced glycation end products (AGEs) [87]. AGE-modified collagen increases matrix stiffness and promotes the accumulation of ECM proteins [88]. The buildup of AGEs is further influenced by ROS accumulation, which is driven by a hypoxic environment [89]. Overall, increased matrix stiffness resulting from AGE accumulation may affect the mechanical regulation of quiescence. Although, the activation of AGE-RAGE signaling has previously been shown to induce cell death to balance tissue homeostasis [89], this could potentially also pertain to both PMF and SF, wherein genes involved in AGE-RAGE signaling were highly expressed compared to PF (Fig. 4C). In addition, it is also important to consider other key factors that may undergo dynamic changes to maintain ovarian function. Previous reports have demonstrated a critical role of zinc in enhancing meiotic competence in in vitro growing oocytes from bovine early antral follicles [90] and its deficiency leading to apoptosis in bovine mural GCs [91]. Recent mouse studies have shown that zinc also plays an important role in preantral folliculogenesis. During this phase, cellular zinc homeostasis is maintained by both *SLC39* family zinc importers and *SLC30* family zinc exporters, and is necessary for conducting biological processes, including the cell cycle and meiosis [92]. When localized to mouse preantral follicles, the expression of *SLC39* family zinc importers and *SLC30* zinc exporters was found to increase from PMF to PF to SF in oocytes, leading to an increase in total zinc content, unlike the variable expression in the GCs [93]. In this bovine dataset, the SF versus PF pairwise comparison revealed an upregulation of zinc importer *SLC39A9* in the SF (Fig. 2B). In contrast, the zinc exporter gene *SLC30A3* was downregulated in the SF. The opposing expression levels of zinc importers and exporters in the PF to SF transition in this dataset suggest a role of zinc in bovine preantral folliculogenesis, thereby extending its known functional relevance.

These mechanisms may act together to maintain homeostasis by preventing excessive cell death and promoting follicle survival, activation, and proliferation [86, 94, 95]. Interestingly, in Group 2 (Fig. 6), pathway analysis revealed focal adhesion and ECM-receptor interaction, PI3K-Akt, AGE-RAGE, and relaxin, which share common collagen genes. Collagen I and IV were predominant, showing different expression patterns across the three follicular stages. Collagen I is the most prevalent type of collagen and is essential for the structural integrity of various tissues. It is present in nearly all connective tissues [96], and surrounding PMF [97]. Collagen I is associated with Collagen XIV [98]. Collagen XIV was described in a study on fibroblast differentiation as the protein responsible for maintaining cell quiescence and is absent during cell differentiation and proliferation [99]. Due to its structural resistance, we hypothesize that it helps maintain quiescence by imposing mechanical force on the PMF [97, 98]. Since collagen must maintain the follicles' quiescent state for an extended period, physiological mechanisms of collagen homeostasis are necessary. *MMP-2* (matrix metallopeptidase 2) is upregulated in PMF, along with Collagen I, suggesting a role in its turnover via lysosomal degradation [100, 101]. As Collagen I decreases, Collagen IV expression rises in SF compared to PF. Located in the basement membrane, Collagen IV may facilitate SF migration toward the medullary region and reinforce follicular architecture, adapting to continuous ECM remodeling during follicle growth and luteinization [98, 102].

During the PMF to PF transition, the candidate central hub gene *FN1* was localized to granulosa cells of small follicles in porcine matrisome studies [103]. Its product, fibronectin, is an essential ECM protein that facilitates intercompartmental crosstalk and cell adhesion [104]. Together with collagen, elastin, and laminin, fibronectin has been studied to map architectural changes in the ovarian matrix during PMF activation, underscoring the role of the microenvironment and mechanobiology in regulating PMF development [105].

In the PF versus SF comparison, the number of DEGs was higher than in the PF versus PMF, consistent with prior observations in sheep preantral follicles [39]. Most enriched processes reflected increased transcriptional activity, ribosome biogenesis, and mitotic cell division during the PF to SF transition, in agreement with known follicular physiology [68, 106–108].

The PF stage may be marked by an initiation phase of mitosis in granulosa cells, driven by upregulation of cell-cycle genes, including cyclins, checkpoint genes, APC/C component *ANAPC7*, and BUB3 and BUB1B [109]. In PF, compared to SF, nucleoporins, *NUP133*,* NUP155*,* NUP37*,* NUP43*,* NUP54*,* NUP88*,and* NUP93*, are upregulated, reflecting a possible role in maintaining the nuclear architecture, regulate transcription, and mitosis [110]. Interestingly, the homologous recombination (HR) pathway was enriched in PF compared with SF, indicating DNA repair and transcriptional reactivation in growing follicles [68, 83]. Additionally, pathways related to cell communication, including gap junctions, focal adhesions, Wnt and Notch signaling, were also enriched. The upregulation of gap junction genes at the SF stage likely reflects enhanced intercellular coordination required to maintain meiotic arrest and synchronize oocyte-granulosa interactions, consistent with previous findings in advanced follicle stages [111–114].

Relaxin signaling was also enriched across both transitions, consistent with its reported presence in early human folliculogenesis [115]. During the PF to SF transition, additional enrichment of PI3K-Akt, ECM-receptor interactions, and focal adhesion pathways highlights coordinated regulation of ECM remodeling and intercellular communication [116].

An interesting finding emerged in Group 1 (Fig. 6), where a similar expression pattern was observed for genes enriched in HR, cell cycle regulation, and cellular senescence mechanisms in both PMF and PF. DNA repair mechanisms are tightly coordinated with cell cycle progression through the activation of orchestrated signaling pathways known as DNA damage checkpoints, such as *CHEK1*. When DNA damage is not repaired, these pathways delay or stop the cell cycle at critical points before or during DNA replication, and before cell division, to avoid copying or segregating damaged DNA [117–119]. HR is the main pathway used during DSB repair, with RAD51 as its key protein [120]. In oocytes, HR and RAD51 are crucial in maintaining genome stability by repairing DNA lesions, since even a single unrepaired DNA break can lead to PMF loss [121]. However, what we observed was not the repair of DNA in the nucleus of preantral follicles, but increased expression of RAD51 in the ooplasm. Knocking down *Rad51* has been shown to cause damage to both nuclear and mitochondrial DNA, impair mitochondrial function, and disrupt progression from MI to MII [122]. Importantly, RAD51 localized in the cytoplasm as did the mitochondrial marker HSP60, rather than with the DNA damage marker γH2AX, indicating a putative role beyond nuclear repair.

Previous studies have reported that RAD51 is primarily localized in the ooplasm of mouse preantral follicles [123] and in the inner mitochondrial matrix [124], where it plays a key role in maintaining mitochondrial DNA integrity under oxidative stress conditions [125]. In ovarian cancer cells, depleting RAD51 leads to CHK1-dependent G2/M arrest, mitochondrial accumulation, and increased oxidative stress, which collectively suppress tumor cell proliferation. Therefore, nuclear DNA damage and mitochondrial oxidative stress create a vicious cycle that underlies the tumor-suppressive effect of RAD51 inhibition [126]. In oocytes, however, oxidative stress caused by reactive oxygen species (ROS) can be particularly harmful due to their prolonged lifespan in the ovary. Adaptive mechanisms in primordial oocytes, such as suppression of Complex I, represent strategies that promote longevity while maintaining biological activity [127]. At more advanced developmental stages, RAD51 has been reported to play novel roles in mitochondrial function during in vitro oocyte maturation. In studies using RAD51 depletion or inhibition in mice or porcine oocytes, RAD51 deficiency was associated with abnormal spindle assembly, disrupted mitochondrial distribution, and reduced mitochondrial number [122, 128]. Overall, based on our findings we hypothesize that RAD51 plays a dual role in preantral follicles: traditionally recognized for its role in nuclear DNA repair, it may also help maintain mitochondrial homeostasis and redox balance, which are vital for the long-term survival and integrity of these long-lived ovarian cells. However, this compelling hypothesis still awaits thorough functional validation.

## Conclusions

**Fig. 9:**
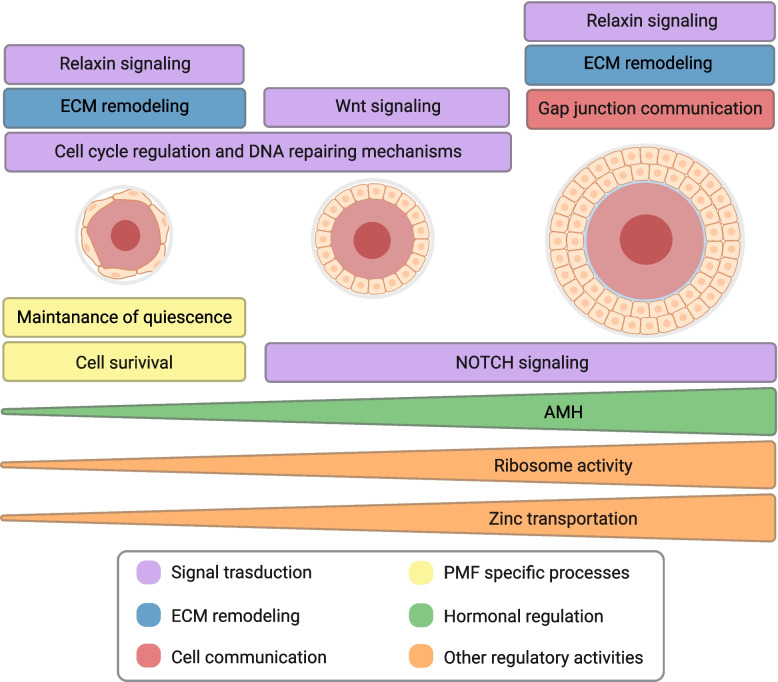
Distinct molecular programs guiding bovine preantral follicle transitions. Graphical representation of the mechanisms highlighted by the transcriptome analysis of PMF, PF, and SF. The mechanisms are grouped based on the color as shown in the legend in the bottom of the figure. Created in https://BioRender.com

In summary, our integrative transcriptomic analysis delineates distinct molecular programs guiding bovine preantral follicle transitions (Fig. 9). By characterizing stage-specific pathways, hub genes, and molecular networks, we provide a foundational framework for understanding follicular development in large mammals.

This study provides the first comprehensive transcriptomic dataset of bovine preantral follicles, offering a detailed characterization of gene expression dynamics across PMF, PF, and SF stages and serving as a valuable hypothesis-generating resource. Some limitations should be explicitly acknowledged. Follicles were sequenced in bulk because the physical separation of oocyte and granulosa cell compartments is technically demanding and is known to compromise cell survival; once isolated, especially after enzymatic digestion, these small follicles and their cellular components rapidly lose viability and may undergo cell death, as previously reported [47, 129, 130]. In addition, the variable number of follicles pooled per replicate and the stage-dependent changes in the oocyte-to-granulosa cell ratio may have influenced measured gene abundance, although normalization procedures were applied to reduce this effect. As livestock research enters the omics era [131], the integration of single-cell and purified-compartment approaches will enable higher-resolution dissection of compartment-specific dynamics and biological processes.

Overall, this work advances current knowledge of folliculogenesis and opens new avenues for future investigations, including spatial and functional validation with potential physiological relevance for the development of effective in vitro follicle culture systems. The dataset also represents a valuable resource for cross-species comparisons and applications in reproductive biotechnology.

## Supplementary Information

Additional file 1: Fig. S1. PCA plot of all sequenced samples, before removing the outlying PF, 47PF00.

Additional file 2: Table S1A. Differentially expressed genes in PF versus PMF resulting from analysis with DESeq2 (*P*adj < 0.05). Table S1B. Functional enrichment Gene Ontology Biological Processes of differentially expressed genes in PF versus PMF. Table S1C. Functional enrichment KEGG pathway analysis of differentially expressed genes in PF versus PMF. Table S1D. Differentially expressed genes in SF versus PF resulting from analysis with DESeq2 (*P*adj < 0.05). Table S1E. Functional enrichment Gene Ontology Biological Processes of differentially expressed genes in SF versus PF. Table S1F. Functional enrichment KEGG pathway analysis of differentially expressed genes in SF versus PF.

Additional file 3: Table S2A. List of transcription factors mapped to the DEGs identified in the PF versus PMF comparison. Of the TF, 12 were found to be genes differentially expressed in this analysis as indicated in Fig. 4A. Table S2B. List of transcription factors mapped to the DEGs identified in the SF versus PF comparison. Of the TF, 68 were found to be genes differentially expressed in this analysis as indicated in Fig. 5A.

Additional file 4: Fig. S2. Hub gene subnetworks of DEGs between PF versus PMF obtained through (a) Degree Centrality, (b) Betweenness Centrality, (c) Closeness Centrality and (d) Eigen vector Centrality. Fig. S3. Hub gene subnetworks of DEGs between SF versus PF obtained through (a) Degree Centrality, (b) Betweenness Centrality, (c) Closeness Centrality and (d) Eigen vector Centrality.

Additional file 5: Fig. S4. Negative control of immunohistochemistry in the bright field conducted with the omission of the primary antibody. Fig. S5A. Negative control of immunofluorescence conducted with the omission of the primary antibody RAD51 while retaining both secondary antibodies. Fig. S5B. Negative control of immunofluorescence conducted with the omission of the primary antibody ɣH2AX while retaining both secondary antibodies. Fig. S5C. Negative control of immunofluorescence conducted with the omission of the primary antibody RAD51 while retaining both secondary antibodies. Fig. S5D. Negative control of immunofluorescence conducted with the omission of the primary antibody HSP60 while retaining both secondary antibodies.

## Data Availability

The datasets generated and analyzed during the current study are available in the Gene Expression Omnibus (GEO) repository, GSE283263 and GSE309360.

## Abbreviations

AMH: Anti-Müllerian hormone
ARTs: Assisted reproductive technologies
BP: Biological processes
BSA: Bovine serum albumin
DEGs: Differentially expressed genes
DGE: Differential gene expression
DSB: Double-strand break
ECM: Extracellular matrix
FDR: False discovery rate
GO: Gene Ontology
HR: Homologous recombination
log_2_FC: Log_2_ Fold Change
LRT: Likelihood ratio test
NGS: Next-generation sequencing
ORA: Over representation analysis
PCA: Principal component analysis
PF: Primary follicle
PMF: Primordial follicle
rlog: Log-transformed counts
ROS: Reactive oxygen species
SD: Standard deviation
SF: Secondary follicle
TF: Transcription factors
