# Supplementary figures and images for: Transcriptome profiling of bovine preantral follicles during early folliculogenesis
Source: J Anim Sci Biotechnol. 2026 May 14;17:92. doi: 10.1186/s40104-026-01407-w (PMC13173735; doi:10.1186/s40104-026-01407-w)

**Additional file 1**

[Fig. S1]

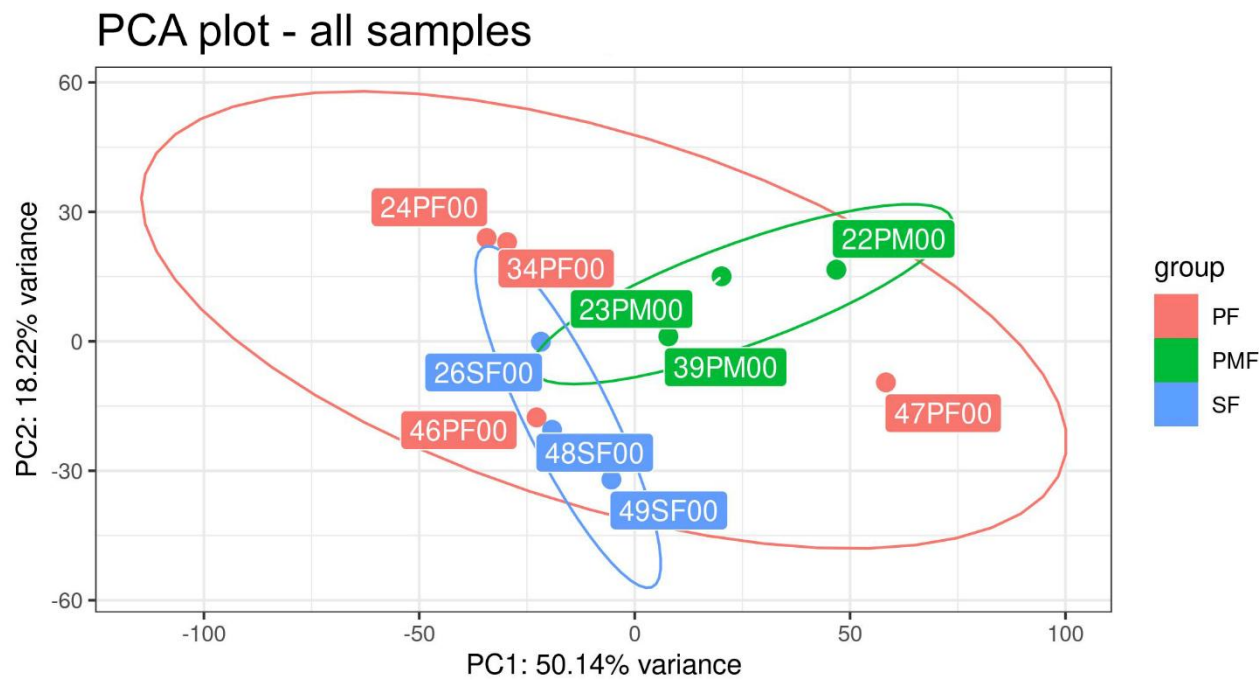

Fig. S1 PCA plot of all sequenced samples, before removing 47PF00.

Supplement: Supplementary file 1 — Additional file 1: Fig. S1. PCA plot of all sequenced samples, before removing the outlying PF, 47PF00. [file 40104_2026_1407_MOESM1_ESM.pdf]
