# Supplementary material for: Transcriptome profiling of bovine preantral follicles during early folliculogenesis
Source: J Anim Sci Biotechnol. 2026 May 14;17:92. doi: 10.1186/s40104-026-01407-w (PMC13173735; doi:10.1186/s40104-026-01407-w)
Supplement: Supplementary file 4 — Additional file 4: Fig. S2. Hub gene subnetworks of DEGs between PF versus PMF obtained through (a) Degree Centrality, (b) Betweenness Centrality, (c) Closeness Centrality and (d) Eigen vector Centrality. Fig. S3. Hub gene subnetworks of DEGs between SF versus PF obtained through (a) Degree Centrality, (b) Betweenness Centrality, (c) Closeness Centrality and (d) Eigen vector Centrality. [file 40104_2026_1407_MOESM4_ESM.pdf]

Additional file 4

[Fig. S2]

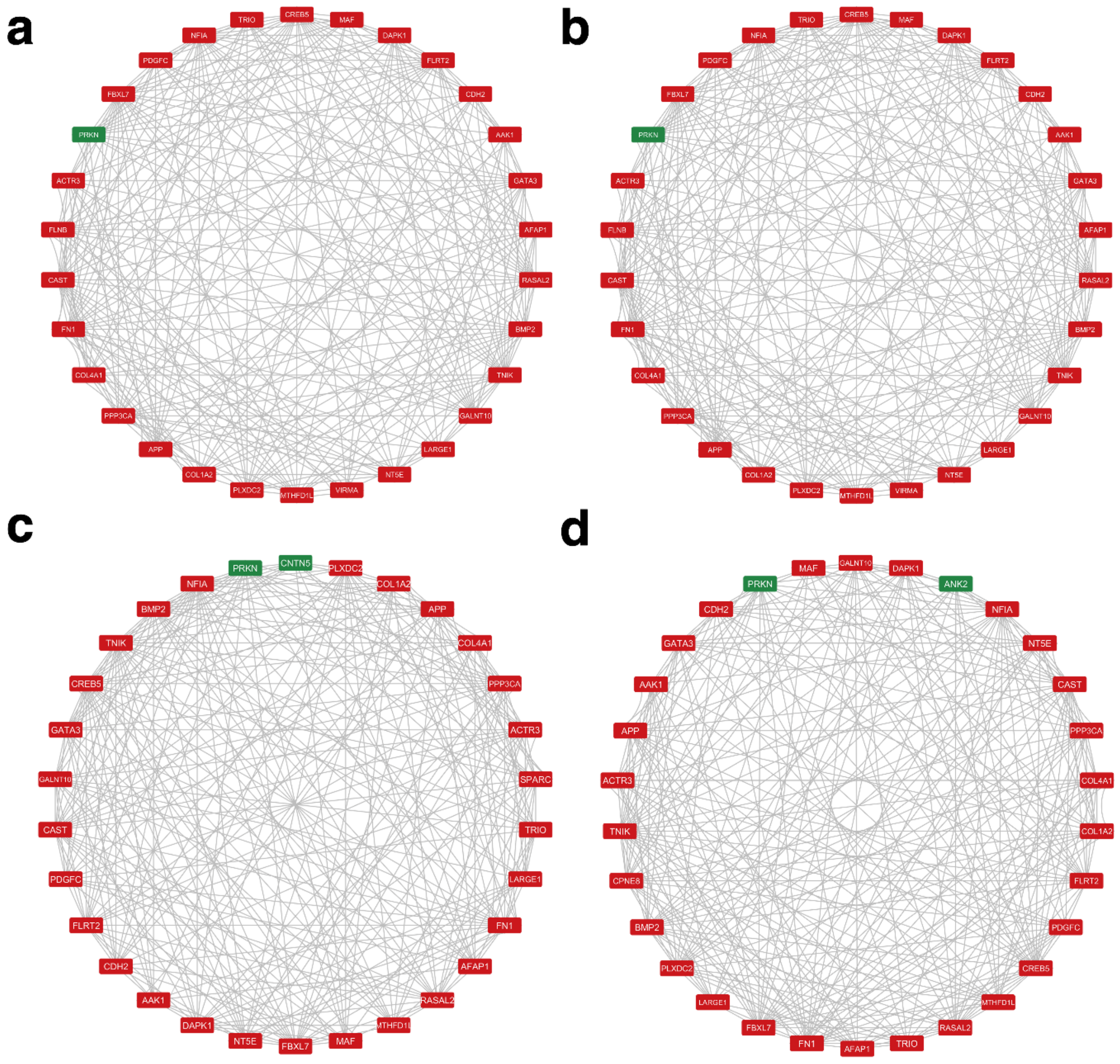

Fig. S2: Hub gene subnetworks of DEGs between PF versus PMF obtained through (a) Degree Centrality, (b) Betweeness Centrality, (c) Closeness Centrality and (d) Eigen vector Centrality.

[Fig. S3]

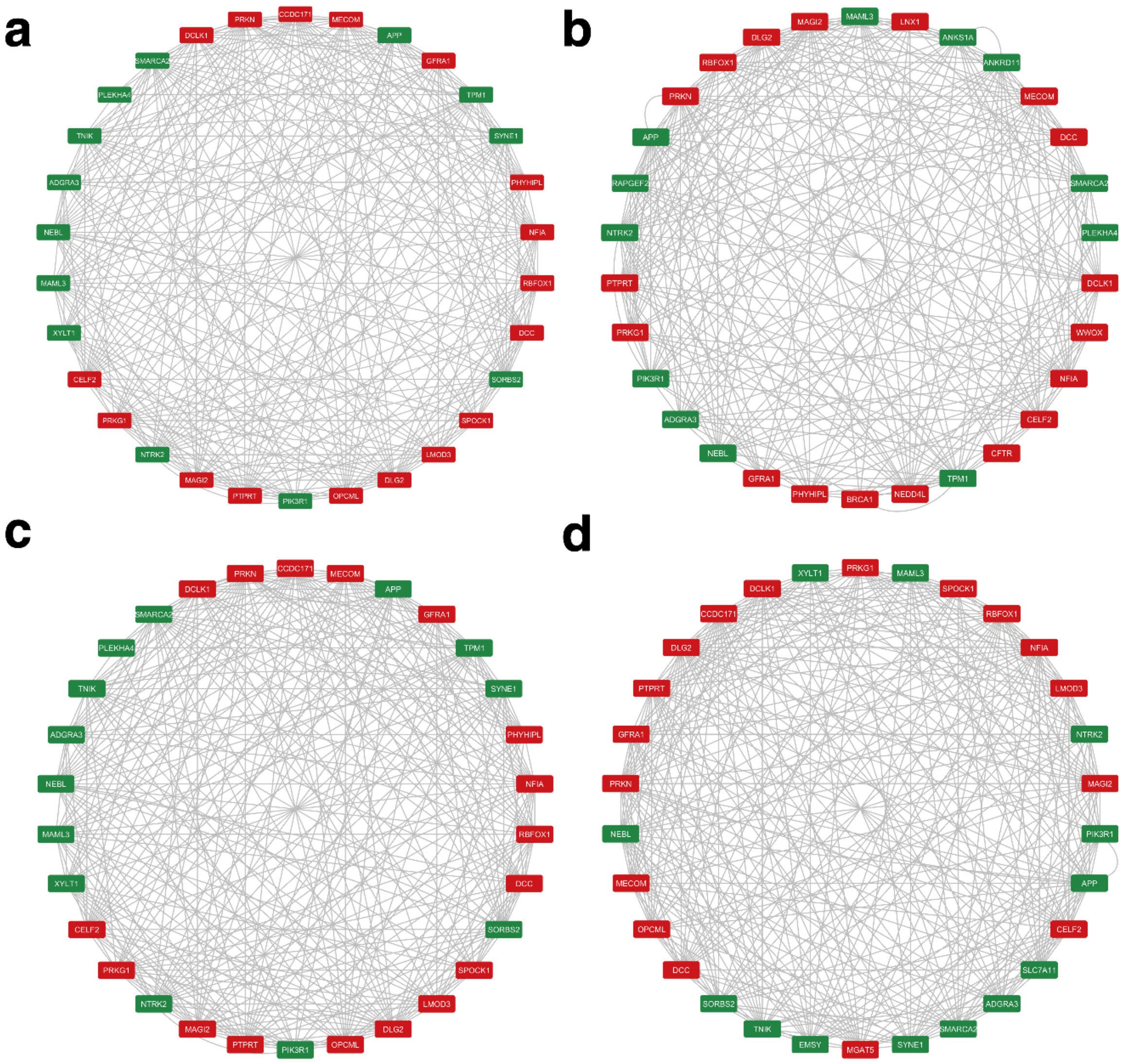

Fig. S3: Hub gene subnetworks of DEGs between SF versus PF obtained through (a) Degree Centrality, (b) Betweeness Centrality, (c) Closeness Centrality and (d) Eigen vector Centrality.
