# Supplementary material for: Transcriptome profiling of bovine preantral follicles during early folliculogenesis
Source: J Anim Sci Biotechnol. 2026 May 14;17:92. doi: 10.1186/s40104-026-01407-w (PMC13173735; doi:10.1186/s40104-026-01407-w)
Supplement: Supplementary file 5 — Additional file 5: Fig. S4. Negative control of immunohistochemistry in the bright field conducted with the omission of the primary antibody. Fig. S5A. Negative control of immunofluorescence conducted with the omission of the primary antibody RAD51 while retaining both secondary antibodies. Fig. S5B. Negative control of immunofluorescence conducted with the omission of the primary antibody ɣH2AX while retaining both secondary antibodies. Fig. S5C. Negative control of immunofluorescence conducted with the omission of the primary antibody RAD51 while retaining both secondary antibodies. Fig. S5D. Negative control of immunofluorescence conducted with the omission of the primary antibody HSP60 while retaining both secondary antibodies. [file 40104_2026_1407_MOESM5_ESM.pdf]

Additional file 5

[Fig. S4]

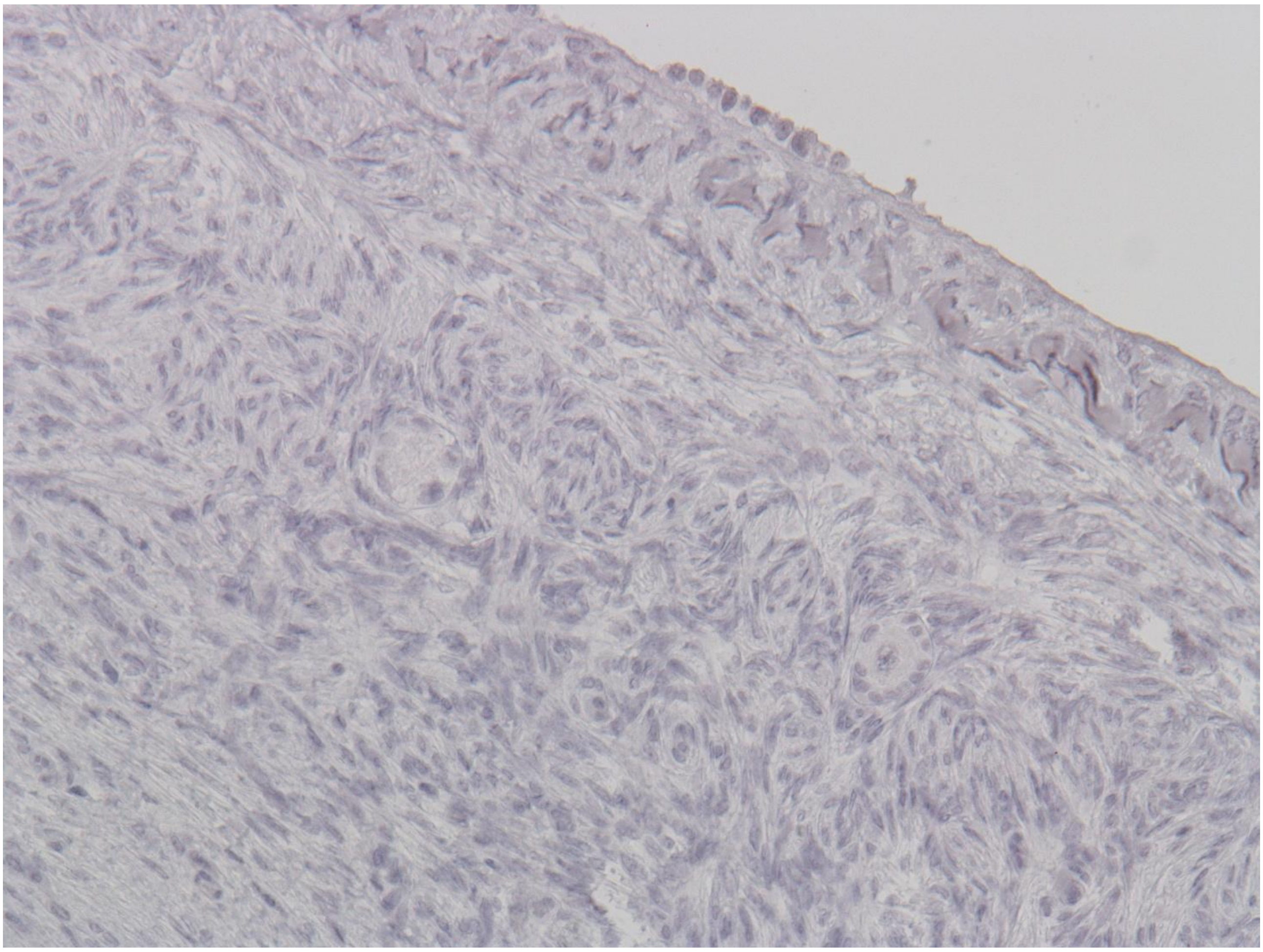

Fig. S4 Negative control of immunohistochemistry in the bright field conducted with the omission of the primary antibody.

[Fig. S5]

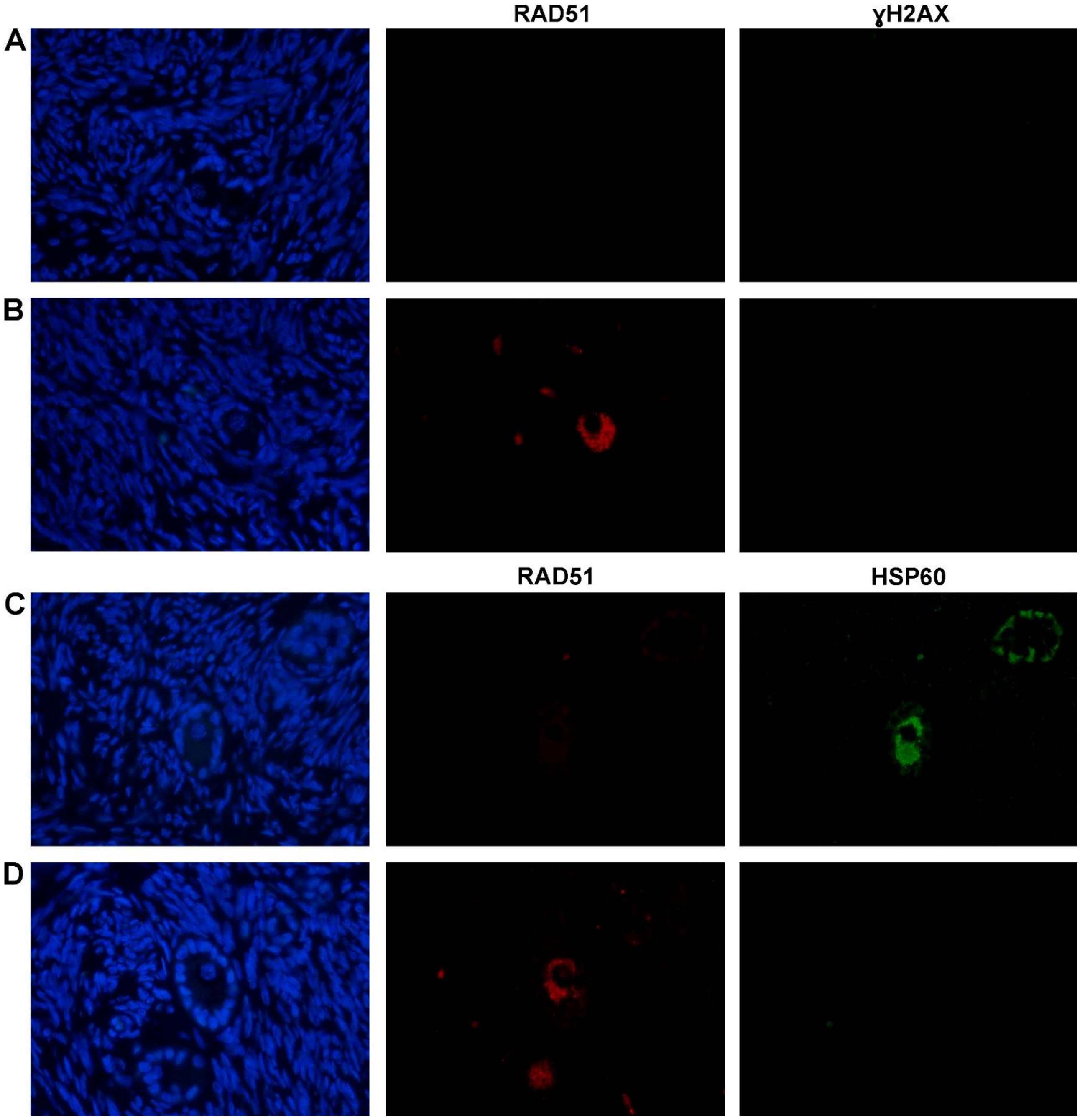

Fig.S5A Negative control of immunofluorescence conducted with the omission of the primary antibody RAD51 while retaining both secondary antibodies. Fig. S5B Negative control of immunofluorescence conducted with the omission of the primary antibody ɣH2AX while retaining both secondary antibodies. Fig.S5C Negative control of immunofluorescence conducted with the omission of the primary antibody RAD51 while retaining both secondary antibodies. Fig. S5D Negative control of immunofluorescence conducted with the omission of the primary antibody HSP60 while retaining both secondary antibodies.
